# Panniculectomy With Umbilical Transposition in the Era of Glucagon-Like Peptide-1 Receptor Agonists: The Impact of Weight Loss Modality on Postoperative Outcomes

**DOI:** 10.1093/asjof/ojag206

**Published:** 2026-10-15

**Authors:** Kasra N Fallah, Parhom N Towfighi, Louis Massoud, Angad S Sidhu, Dillon C Mobasser, Aniffa S Kouton, Ryan D Harmelink, Nikhi P Singh, George S Corpuz, Ravinder Bamba, Rachel M Danforth, Richard J VonDerHaar, Mary E Lester, Aladdin H Hassanein, Ivan Hadad

## Abstract

**Background:**

Panniculectomy is performed to address excess infraumbilical skin and soft tissue following weight loss. Weight loss can be achieved through lifestyle modifications, bariatric surgery, and pharmacologic therapies, such as glucagon-like peptide-1 (GLP-1) agonists. The impact of weight loss modality on postoperative outcomes remains unclear.

**Objectives:**

The aim of this study was to evaluate whether the method of weight loss influences postoperative outcomes following panniculectomy.

**Methods:**

A single-center retrospective review was conducted of patients who underwent panniculectomy between 2021 and 2025. Patients were grouped into 4 cohorts: lifestyle modifications alone, bariatric surgery, GLP-1 agonist therapy, or combined bariatric surgery and GLP-1 agonist therapy. Patients receiving abdominoplasty with extensive undermining and rectus plication were excluded. Variables of interest included patient demographics, weight loss history, comorbidities, wound-healing complications, surgical-site infection, hematoma/seroma, and hospital utilization. Multivariate logistic regression was performed to analyze postoperative complications and healthcare utilization, with statistical significance set at *P* < .05.

**Results:**

A total of 281 patients met inclusion criteria (lifestyle modifications: *n* = 53 [18.9%]; bariatric surgery: *n* = 184 [65.5%]; GLP-1 agonist therapy: *n* = 20 (7.1%); and combined bariatric surgery and GLP-1 agonist therapy: *n* = 24 [8.5%]). Multivariate analysis showed no statistically significant differences in postoperative outcomes, including wound complications, surgical-site infections, seroma/hematoma, or hospital utilization.

**Conclusions:**

Postoperative outcomes following panniculectomy may be similar regardless of weight loss method. Preoperative GLP-1 agonist therapy, alone or combined with bariatric surgery, was not associated with increased postoperative complications or healthcare utilization. Our results can help guide risk stratification and decision making for individuals pursuing panniculectomy after weight loss.

**Level of Evidence: 3 (Risk):**

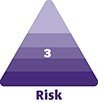

Obesity remains a significant public health issue, with rising prevalence and an increasing burden of associated comorbidities.^1,2^ Multiple conditions are associated with obesity, including hypertension (HTN), type 2 diabetes, obstructive sleep apnea, and cardiovascular disease.^3^ In addition to physical ailments, obesity is linked to multiple mental health and mood disorders, including depression.^4^ With an alarming increase in obesity over the years, national and global efforts have focused on addressing the epidemic through both nonoperative and operative interventions. Many strategies have proved to be highly effective, allowing patients to achieve significant weight loss.

Weight loss strategies include lifestyle changes, bariatric surgery, and pharmacologic treatments, such as glucagon-like peptide-1 (GLP-1) receptor agonists. Current evidence indicates that lifestyle interventions alone can result in 5% to 10% weight loss, GLP-1 agonists can achieve 8% to 21% weight loss, and bariatric surgery can achieve 25% to 30% weight loss.^3,5^ The high efficacy of these approaches, especially the recent increase in GLP-1 receptor agonist usage, aligns with the increasing patient demand for body contouring surgeries. Body and extremity plastic surgery procedures have increased by 96% between 2009 and 2023.^6^

One of the most commonly performed body contouring procedures is panniculectomy, which addresses the infraumbilical pannus. This is a major contributor to poor abdominal aesthetics and is a source for functional limitations, including impaired hygiene, intertrigo, and reduced mobility. However, complication rates with panniculectomy can be variable, with some studies reporting as high as 56%.^7^ Panniculectomy after bariatric surgery has demonstrated wound dehiscence rates of 24%, seroma rates of 18%, and surgical-site infection rates of 22%.^8^

Weight loss modalities may result in varying physiologic effects, which can potentially influence surgical outcomes after body contouring procedures. However, there is a paucity of literature comparing panniculectomy outcomes across different methods of weight loss. The aim of this study is to evaluate whether the method of weight loss influences postoperative outcomes following panniculectomy in patients after weight loss.

## METHODS

A single-center, retrospective review that was approved by the IRB (#27664) was conducted to identify all adult patients who underwent panniculectomy between 2021 and 2025. These patients had previously been referred to our plastic surgery team for evaluation for panniculectomy after weight loss. Standard selection criteria for panniculectomy included a symptomatic pannus (chronic intertrigo, skin breakdown, functional impairment, etc), weight stability, and adequate optimization of medical comorbidities. At our institution, panniculectomies include transposition of the umbilicus but do not include rectus plication. Skin resection is carried to the level of the umbilicus, and undermining of the abdominal skin flap is limited to a central strip no greater than 5 cm. As such, the extent of the flap elevation and dead space creation closely approximates that of a traditional infraumbilical panniculectomy. The panniculectomies were performed by 6 different plastic surgeons, and there were no significant changes to surgical technique or perioperative management protocols over the study period.

A total of 378 patients were identified. Patients were stratified into the following cohorts based on preoperative weight loss methods: lifestyle modifications alone (ie, diet and exercise; LM-only cohort), bariatric surgery (ie, sleeve gastrectomy, gastric bypass, and gastric band; BS-only cohort), GLP-1 agonist therapy (GLP-1-only cohort), and combined bariatric surgery and GLP-1 agonist therapy (GLP-1 + BS cohort). Classification of weight loss was determined through review of clinical notes, operative reports, medication records, and documented weight trajectories within the electronic medical record. There were 97 patients who were excluded from our study because they either underwent panniculectomy without a history of weight loss or underwent abdominoplasty, a procedure not routinely offered at our institution given the lack of insurance coverage for rectus plication.

Patient demographics, history of weight loss, comorbidities, and smoking status were evaluated. Preoperative albumin and prealbumin were also captured to assess nutritional status. Postoperative variables of interest included seroma/hematoma, any wound-related complication (wound dehiscence and surgical-site infection), need for revision surgery, need for postoperative antibiotic use, 30-day emergency department (ED) visits, 30-day postoperative admission, hospital length of stay, and drain duration. Wound-healing complications were further stratified into 3 cohorts based on severity: need for outpatient local wound care alone, need for nonsurgical debridement, or need for surgical debridement. Infectious complications were further stratified into 3 cohorts based on severity: need for outpatient antibiotics alone, need for hospital admission for intravenous antibiotics, or need for surgical intervention.

Baseline characteristics were compared across groups using 1-way analysis of variance for continuous variables and χ^2^ or Fisher's exact tests for categorical variables. Univariate logistic regression was performed between weight loss method and each postoperative outcome. Multivariate logistic regression models were then constructed to evaluate independent associations between weight loss methods and postoperative outcomes. The following covariates were adjusted for: age, BMI at time of surgery, diabetes, smoking status, American Society of Anesthesiologists class, total weight loss, chronic obstructive pulmonary disease, and venous thromboembolism (VTE). All statistical analyses were performed using IBM SPSS Statistics (v.31.0.0, IBM Corp., Armonk, NY). Statistical significance was defined as a *P*-value <.05.

## RESULTS

A total of 281 patients met our inclusion criteria (LM-only cohort: 53 [18.9%], BS-only cohort: 184 [65.5%], GLP1-only cohort: 20 [7.1%], GLP1 + BS cohort: 24 [8.5%]). The mean age was 48.5 years, with a range of 18 to 77 years. Of these patients, 247 (87.9%) were female and 34 (12.1%) were male. Baseline preoperative characteristics, including age, sex, race, insurance status, marital status, BMI at start of weight loss journey, and duration of weight loss, were similar across cohorts (Table 1). BMI at the time of surgery was highest in the LM-only cohort and lowest in the GLP1 + BS cohort (36.5 ± 9.5 kg/m^2^ vs 31.4 ± 6.1 kg/m^2^; *P* = .045). Total weight loss was highest in the GLP1 + BS cohort and lowest in GLP-only cohort (60.8 ± 36.0 kg vs 40.9 ± 20.6 kg; *P* = .002). Patient comorbidities, including HTN, hemoglobin A1c, obstructive sleep apnea, coronary artery disease, psychiatric history, smoking, alcohol use, illicit substance use, albumin level, and prealbumin level, were not significantly different across groups. Prevalence of diabetes was highest in the GLP1-only cohort and lowest in the LM-only cohort (75% vs 21%; *P* < .001).

**Table 1.** Baseline Characteristics Stratified by Method of Weight Loss

Characteristic	Lifestyle mod. (*n* = 53)	Bariatric only (*n* = 184)	GLP-1 only (*n* = 20)	GLP-1 + Bariatric (*n* = 24)	*P*-value*
Age (years), mean ± SD	47.2 ± 14.4	48.0 ± 12.1	53.9 ± 13.4	49.7 ± 10.6	.192
Female, *n* (%)	42 (79)	164 (89)	19 (95)	22 (92)	.155
Race, *n* (%)					.683
White	35 (66)	119 (65)	12 (60)	13 (54)	
Black	15 (28)	58 (32)	8 (40)	9 (38)	
Other	3 (5.7)	7 (3.8)	0 (0)	2 (8.3)	
Marital status, *n* (%)					.824
Married	18 (34)	69 (38)	9 (45)	8 (33)	
Unmarried/divorced/widowed	35 (66)	115 (63)	11 (55)	16 (67)	
Insurance type, *n* (%)					.595
Commercial	17 (32)	48 (26)	9 (45)	8 (33)	
Medicaid	21 (40)	76 (41)	4 (20)	7 (29)	
Medicare	6 (11)	16 (8.7)	5 (25)	4 (17)	
Self-pay/uninsured	8 (15)	44 (24)	2 (10)	5 (21)	
Other	1 (1.9)	0 (0)	0 (0)	0 (0)	
Diabetes, *n* (%)	11 (21)	44 (24)	15 (75)	12 (50)	<.001*
HbA1c (%), mean ± SD	5.59 ± 0.8	5.71 ± 1.0	5.89 ± 1.0	6.14 ± 1.0	.619
HTN, *n* (%)	22 (42)	85 (46)	13 (65)	16 (67)	.079
OSA, *n* (%)	9 (17)	53 (29)	9 (45)	6 (25)	.100
CAD, *n* (%)	2 (3.8)	9 (4.9)	4 (20)	2 (8.3)	.058
COPD, *n* (%)	0 (0)	5 (2.7)	1 (5.0)	3 (13)	.038*
Previous VTE, *n* (%)	7 (13)	10 (5.4)	4 (20)	1 (4.2)	.041*
Psychiatric history, *n* (%)	23 (43)	91 (49)	8 (40)	15 (63)	.384
Smoking, *n* (%)	7 (13)	11 (6.0)	3 (15)	3 (13)	.207
Alcohol use, *n* (%)	22 (42)	73 (40)	8 (40)	8 (33)	.925
Illicit substance use, *n* (%)	5 (9.4)	5 (2.7)	0 (0)	0 (0)	.062
Albumin (g/dL), mean ± SD	4.00 ± 0.4	4.06 ± 0.3	4.23 ± 0.3	4.13 ± 0.4	.317
Prealbumin (mg/dL), mean ± SD	27.0 ± N/A	24.8 ± 6.1	30.0 ± 2.8	20.7 ± 2.2	.239
BMI at start of weight loss (kg/m^2^), mean ± SD	51.3 ± 13.2	53.8 ± 11.2	50.7 ± 10.8	54.0 ± 16.0	.453
BMI at time of surgery (kg/m^2^), mean ± SD	36.5 ± 9.5	33.8 ± 8.1	35.4 ± 6.0	31.4 ± 6.1	.045*
Total weight loss (kg), mean ± SD	44.8 ± 33.9	58.2 ± 25.0	40.9 ± 20.6	60.8 ± 36.0	.002*
Weight loss time (months), mean ± SD	30.3 ± 18.0	86.1 ± 89.7	50.0 ± 32.6	97.0 ± 106.9	.194
Concurrent procedure, *n* (%)	7 (13)	19 (10)	2 (10)	3 (13)	.936
ASA class, *n* (%)					.697
1	2 (3.8)	3 (1.7)	0 (0)	0 (0)	
2	21 (40)	92 (51)	9 (45)	14 (58)	
3	28 (54)	84 (47)	11 (55)	10 (42)	
4	1 (1.9)	1 (0.6)	0 (0)	0 (0)	
Outpatient, *n* (%)	37 (70)	130 (71)	13 (65)	16 (67)	.943

Data presented as mean ± SD or *n* (%) as indicated.

ASA, American Society of Anesthesiologists; CAD, coronary artery disease; COPD, chronic obstructive pulmonary disease; GLP-1, glucagon-like peptide-1; HbA1c, hemoglobin A1c; HTN, hypertension; OSA, obstructive sleep apnea; SD, standard deviation; VTE, venous thromboembolism.

^*^ *P* < .05.

There were no statistically significant differences in the rates of any postoperative wound complication between all cohorts (LM-only cohort: 43%, BS-only cohort: 32%, GLP1-only cohort: 45%, GLP1 + BS cohort: 50%; *P* = .134; Table 2). Median follow-up was just over 100 days, and this was not significantly different between the groups. Hospital length of stay, drain duration, and BMI at most recent follow-up were also not significantly different between cohorts. In univariate analysis, using the lifestyle modifications cohort as the reference group, no weight loss modality was significantly associated with overall wound complications, seroma/hematoma, wound dehiscence, surgical-site infection, ED visit within 30 days, readmission within 30 days, revision surgery, postoperative oral antibiotic use, intravenous antibiotic use, local wound care, nonsurgical debridement, surgical debridement, or surgical washout (Table 3). These findings remained without statistical significance after multivariate logistic regression (Table 4).

**Table 2.** Unadjusted Postoperative Outcomes Stratified by Method of Weight Loss

Outcome	Lifestyle mod. (*n* = 53)	Bariatric only (*n* = 184)	GLP-1 only (*n* = 20)	GLP-1 + Bariatric (*n* = 24)	*P*-value
Any wound complication, *n* (%)	23 (43)	58 (32)	9 (45)	12 (50)	.134
Seroma/hematoma, *n* (%)	12 (23)	24 (13.0)	1 (0.5)	7 (29.2)	.580
Wound dehiscence, *n* (%)	12 (23)	31 (17)	8 (40)	6 (25)	.083
SSI, *n* (%)	11 (21)	24 (13)	5 (25)	3 (13)	.324
RTOR, *n* (%)	9 (17)	15 (8.2)	0 (0)	4 (17)	.070
Local wound care, *n* (%)	13 (25)	25 (13)	4 (20)	5 (21)	.255
Nonsurgical debridement, *n* (%)	4 (7.0)	8 (4.3)	2 (10.0)	1 (4.2)	.645
Surgical debridement, *n* (%)	1 (1.8)	6 (3.2)	1 (5.0)	2 (8.3)	.501
Chronic wound, *n* (%)	0 (0)	3 (1.6)	0 (0)	0 (0)	.656
Wound referral, *n* (%)	4 (7.5)	9 (4.8)	3 (15.0)	3 (12.5)	.214
Postoperative oral antibiotics, *n* (%)	15 (28)	34 (19)	7 (35)	4 (17)	.181
Postoperative IV antibiotics, *n* (%)	2 (3.5)	4 (2.2)	1 (5.0)	2 (8.3)	.398
OR washout, *n* (%)	3 (5.4)	7 (3.8)	1 (5.0)	1 (4.2)	.959
ED visit, 30 days, *n* (%)	9 (17)	45 (24)	4 (20)	8 (33)	.428
Readmission, 30 days, *n* (%)	6 (11)	19 (10)	1 (5.0)	5 (21)	.363
VTE, *n* (%)	0 (0)	1 (0.5)	0 (0)	0 (0)	.912
LOS (days), median (IQR)	1.00 (1.00-4.00)	1.00 (1.00-2.00)	3.50 (1.00-7.00)	2.00 (1.50-3.00)	.105
Drain duration (days), median (IQR)	20 (14-28)	18 (13-23)	20 (13-24)	16 (13-22)	.298
Discharge disposition, *n* (%)					.813
Home	51 (96)	180 (98)	19 (95)	23 (96)	
Other facility	2 (3.8)	4 (2.2)	1 (5.0)	1 (4.2)	
Follow-up (days), median (IQR)	103 (49-219)	107 (41-197)	112 (91-218)	113 (91-165)	.434
Recent BMI (kg/m^2^), mean ± SD	36 ± 9	35 ± 30	34 ± 6	31 ± 6	.119
Revision surgery, *n* (%)	8 (15)	21 (11)	3 (15)	3 (13)	.887

Data presented as *n* (%), median (Q1-Q3) (interquartile range), or mean ± SD, as indicated for each row. No outcomes reached statistical significance (*P* < .05); no asterisks appear in this table.

ED, emergency department; GLP-1, glucagon-like peptide-1; IQR, interquartile range; IV, intravenous; LOS, length of stay; OR, operating room; SD, standard deviation; VTE, venous thromboembolism; RTOR, return to operating room; SSI, surgical-site infection.

**Table 3.** Univariate Analysis of Postoperative Outcomes

Outcome	Bariatric only vs lifestyle	GLP-1 only vs lifestyle	GLP-1 + Bariatric vs lifestyle
Any wound complication	0.60 (0.32-1.12), *P* = .110	1.07 (0.38-3.00), *P* = .902	1.30 (0.50-3.43), *P* = .590
Seroma/hematoma	0.58 (0.26-1.33), *P* = .198	0.23 (0.03-1.90), *P* = .171	1.77 (0.58-5.41), *P* = .316
Wound dehiscence	0.69 (0.33-1.47), *P* = .337	2.28 (0.76-6.86), *P* = .143	1.14 (0.37-3.51), *P* = .821
Surgical-site infection	0.58 (0.26-1.27), *P* = .172	1.27 (0.38-4.27), *P* = .696	0.55 (0.14-2.17), *P* = .389
ED visit within 30 days	1.58 (0.72-3.49), *P* = .256	1.22 (0.33-4.53), *P* = .764	2.44 (0.81-7.43), *P* = .115
Readmission within 30 days	0.90 (0.34-2.39), *P* = .836	0.41 (0.05-3.66), *P* = .426	2.06 (0.56-7.57), *P* = .276
Revision	0.73 (0.30-1.75), *P* = .473	0.99 (0.24-4.19), *P* = .992	0.80 (0.19-3.34), *P* = .763
Postoperative oral antibiotics	0.58 (0.29-1.17), *P* = .127	1.36 (0.46-4.08), *P* = .579	0.51 (0.15-1.73), *P* = .278
Local wound care	0.48 (0.23-1.03), *P* = .059	0.77 (0.22-2.72), *P* = .684	0.81 (0.25-2.60), *P* = .723
Nonsurgical debridement	0.56 (0.16-1.93), *P* = .355	1.36 (0.23-8.08), *P* = .734	0.53 (0.06-5.04), *P* = .583
Surgical debridement	1.75 (0.21-14.89), *P* = .607	2.74 (0.16-45.97), *P* = .484	4.73 (0.41-54.87), *P* = .214
Wound referral	0.63 (0.19-2.13), *P* = .458	2.16 (0.44-10.66), *P* = .344	1.75 (0.36-8.51), *P* = .488
OR washout	0.65 (0.16-2.59), *P* = .537	0.86 (0.08-8.79), *P* = .899	0.71 (0.07-7.20), *P* = .772
Postoperative IV antibiotics	0.57 (0.10-3.20), *P* = .523	1.34 (0.12-15.67), *P* = .814	2.32 (0.31-17.52), *P* = .415

Reference group: lifestyle modification (OR = 1.00). No comparisons reached statistical significance (*P* < .05); no asterisks appear in this table.

ED, emergency department; GLP-1, glucagon-like peptide-1; IV, intravenous; OR, odds ratio.

**Table 4.** Multivariate Analysis of Postoperative Outcomes

Outcome	Bariatric only vs lifestyle	GLP-1 only vs lifestyle	GLP-1 + Bariatric vs Lifestyle
Any wound complication	0.62 (0.31-1.26), *P* = .184	1.30 (0.40-4.24), *P* = .667	1.36 (0.44-4.14), *P* = .593
Seroma/hematoma	0.47 (0.20-1.12), *P* = .097	0.21 (0.02-1.90), *P* = .175	1.41 (0.42-4.76), *P* = .580
Wound dehiscence	0.79 (0.34-1.86), *P* = .590	2.55 (0.71-9.21), *P* = .153	0.93 (0.22-3.95), *P* = .917
Surgical-site infection	0.72 (0.29-1.77), *P* = .470	2.10 (0.52-8.52), *P* = .299	0.73 (0.13-4.10), *P* = .722
ED visit within 30 days	1.33 (0.57-3.09), *P* = .513	1.17 (0.29-4.80), *P* = .824	2.02 (0.58-7.08), *P* = .270
Readmission within 30 days	0.72 (0.25-2.04), *P* = .532	0.44 (0.04-4.37), *P* = .484	1.46 (0.31-6.85), *P* = .632
Revision	0.76 (0.28-2.06), *P* = .589	0.89 (0.15-5.39), *P* = .896	1.19 (0.24-5.84), *P* = .833
Postoperative oral antibiotics	0.73 (0.33-1.62), *P* = .440	2.06 (0.58-7.38), *P* = .266	0.92 (0.24-3.63), *P* = .910
Local wound care	0.45 (0.19-1.07), *P* = .069	0.75 (0.18-3.20), *P* = .695	0.61 (0.13-2.85), *P* = .528
Nonsurgical debridement	0.46 (0.09-2.32), *P* = .346	0.84 (0.10-7.19), *P* = .876	0.40 (0.03-5.84), *P* = .503
Surgical debridement	1.69 (0.14-20.49), *P* = .681	1.71 (0.07-42.39), *P* = .743	4.04 (0.21-77.94), *P* = .355
Wound referral	1.60 (0.26-9.89), *P* = .612	2.38 (0.29-19.39), *P* = .417	3.15 (0.24-40.89), *P* = .380
OR washout	0.69 (0.11-4.43), *P* = .691	2.01 (0.09-44.13), *P* = .658	Not estimable^a^
IV antibiotics	0.61 (0.08-4.67), *P* = .631	2.27 (0.12-45.13), *P* = .590	1.20 (0.07-21.01), *P* = .901

Reference group: lifestyle modification (OR = 1.00). No comparisons reached statistical significance (*P* < .05); no asterisks appear in this table.

^a^Odds ratio not estimable in the multivariate model for this subgroup.

ED, emergency department; GLP-1, glucagon-like peptide-1; IV, intravenous; OR, odds ratio.

## DISCUSSION

In this study, we evaluated postoperative outcomes following panniculectomy among patients who achieved weight loss through 4 distinct weight loss modalities: lifestyle modifications, bariatric surgery, GLP-1 receptor agonist therapy, and combined bariatric surgery with GLP-1 therapy. On multivariate analysis, there were no statistically significant differences in rates of surgical complications between groups, including hematoma/seroma, VTE, surgical-site infection, or wound dehiscence. There were also no significant differences in postoperative healthcare utilization, including antibiotic use, ED visits, hospital admissions, or surgical revision. These findings suggest that the method of weight loss does not independently impact postoperative outcomes following panniculectomy in the weight loss population.

Although weight loss improves glucose metabolic parameters, insulin sensitivity, and cardiovascular risk, patients undergoing bariatric surgery are at high risk for malnutrition and micronutrient deficiency, which could result in suboptimal wound healing.^9,10^ Historically, there have been concerns regarding an increased postoperative complication risk in patients undergoing body contouring surgery after bariatric surgery.^11,12^ Previous studies have suggested that hypoalbuminemia and deficiencies in vitamin A, vitamin D, and zinc can impair wound healing, leading to higher complication rates.^12^ However, more recent literature has demonstrated that with appropriate preoperative optimization, bariatric surgery patients can achieve surgical outcomes comparable to nonbariatric patients.^13,14^

Glucagon-like peptide-1 receptor agonists have emerged as effective pharmacologic agents for weight loss in patients with obesity. These agents provide significant weight reduction and improvement in cardiovascular risk factors.^15^ However, their implications in the body contouring population remain incompletely understood. In addition to weight loss, GLP-1 agonists have been associated with reductions in lean body mass, including muscle loss.^16^ The loss of lean body mass may negatively impact wound healing and overall surgical recovery. Consequently, some literature suggests that the use of GLP-1 agonists may lead to surgical-site complications after body contouring procedures.^17^ However, in our study, the use of GLP-1 agonists was not associated with an increased rate of wound-healing complications.

To our knowledge, this is among the first studies to examine the influence of weight loss modality on surgical outcomes after panniculectomy. We identified no statistically significant differences in postoperative outcomes between patients who achieved weight loss through pharmacologic means, including GLP-1 receptor agonists, compared with those who underwent bariatric surgery. This is particularly relevant given concerns that GLP-1 agonists may contribute to reductions in lean body mass and protein intake, which would theoretically impair wound healing.^18,19^ Although the GLP-1-only cohort in our study had a higher prevalence of diabetes compared with the other weight loss cohorts, this did not translate into higher rates of wound complications. This contrasts with previous reports that have suggested increased wound dehiscence rates among diabetic patients treated with GLP-1 receptor agonists.^20^ Despite these concerns, our results do not demonstrate an increased risk of wound complications or healthcare utilization in patients treated with GLP-1 therapy.

These results have important implications for surgical candidacy and perioperative counseling. Because GLP-1 receptor agonists become increasingly prevalent in the management of obesity, plastic surgeons are more frequently encountering patients who have achieved significant weight loss through medical therapy alone or in combination with bariatric surgery.^18^ Emphasis should be placed on individualized risk assessment and preoperative optimization, including nutritional status, weight stability, and comorbidity management.^11^ The absence of differences in complication profiles across our cohorts supports a shift away from weight loss modality as a determinant of surgical risk.

The literature on GLP-1 agonists in the surgical population remains limited, but early data suggest that these agents are not associated with adverse surgical outcomes when appropriately managed.^20-22^ Importantly, our study expands on existing literature by directly comparing multiple weight loss modalities, while adjusting for potential confounding variables through multivariate analysis. This approach aimed to reduce potential bias and allow for a more comprehensive comparison between groups.

This study has limitations. As a retrospective chart review, this study is subject to inherent selection bias and unmeasured confounding. This was also a single-center study, which may limit the generalizability of our findings to other institutions and patient populations. Our definition of panniculectomy, which includes umbilical transposition without rectus plication, may not align with the definition used by other providers. This variability in technique should be considered when interpreting our results. Procedures were performed by multiple surgeons, introducing potential variability in operative technique and perioperative management. Bariatric surgery procedures, including gastric bypass, sleeve gastrectomy, and gastric banding, were analyzed as a single category without stratification by procedure type, which may obscure differences in outcomes associated with specific bariatric operations. Future studies with procedure-stratified cohorts will be important for characterizing the differential impact of specific bariatric procedures on panniculectomy outcomes. Patients in both the bariatric surgery cohort and GLP-1 agonist cohort may have also engaged in lifestyle modifications for weight loss, either before or concurrent with their surgical or medical therapies. Nutritional status was not uniformly quantified, and variability in micronutrient deficiencies may have influenced wound healing and complication risk. The GLP-1 only and combined GLP-1 and bariatric surgery cohorts had a smaller sample size than the bariatric surgery-only cohort, limiting statistical power. Therefore, the absence of statistically significant differences should not be interpreted as evidence of equivalence. Furthermore, specific GLP-1 agent, treatment duration, and dosing intensity were not captured, representing potential sources of heterogeneity that may have influenced surgical outcomes. Future studies with large cohorts will be necessary to better characterize the effect of specific GLP-1 agonists and dosing strategies on panniculectomy outcomes. Additionally, ED visits or complications managed at outside institutions may not have been captured in our data, as well as the possibility of late complications occurring beyond our follow-up window. We also did not assess aesthetic outcomes or patient-reported outcome measures.

## CONCLUSIONS

The method of weight loss, whether through lifestyle modifications, bariatric surgery, GLP-1 receptor agonist therapy, or a combination thereof, did not independently impact postoperative complication rates or healthcare utilization following panniculectomy. Our results indicate that similar postoperative complications are expected regardless of weight loss modality if the patients are appropriately optimized. Because pharmacologic weight loss strategies continue to expand, further prospective, multi-institutional studies are needed to better define their long-term impact on surgical outcomes in the body contouring population.
